# “I Know Hyena. Do you Know Hyena?” Challenges in Interpreter-Mediated Dementia Assessment, Focusing on the Role of the Interpreter

**DOI:** 10.1007/s10823-021-09439-7

**Published:** 2022-03-08

**Authors:** Rozita Torkpoor, Ingrid Fioretos, Birgitta Essén, Elisabet Londos

**Affiliations:** 1 Clinical Memory Research Unit, Department of Clinical Sciences, Lund University, Lund, Sweden; 2 Memory Clinic, Skåne University Hospital, SE-205 02 Malmö, Sweden; 3 International Maternal and Child Health (IMCH), Department of Women’s and Children’s Health, Uppsala University, Uppsala, Sweden

**Keywords:** Assessment, Cognition, Communication, Dementia, Interpreter, Language

## Abstract

Dementia assessment requires functional communication and interaction between healthcare professionals and the patient being assessed. These can be affected by the requirement for an interpreter to communicate with the patient. The purpose of this study was to elucidate the interactions between patient, healthcare professionals and interpreter, focusing on the role of the interpreter and the challenges that may arise in interpreter-mediated dementia assessment. The study had an ethnographic design in which the data consisted of audio and video recordings of 19 dementia assessments conducted in the presence of an interpreter. The data were analyzed using the constant comparative method. The results showed that the interpreter could affect the patient’s performance and results during the dementia assessment. The interpreter could alter the meaning and content of what was communicated, sometimes change information and instructions exchanged between the patient and healthcare professionals, could avoid interpreting everything being said, and occasionally made their own corrections to what was being communicated. This occurred mainly because of the interpreter’s lack of linguistic skills and the interpreter failing to adhere to the ethical guidelines governing their profession. These challenges could also occur when the interpreter was not familiar with the context of dementia assessment. Alterations made by the interpreter to what was being communicated could lead to incorrect evaluation of the patient’s cognitive abilities and health status. This, in turn, may lead to misjudgment of the patient’s remaining resources and symptoms and their required treatment and support.

## Introduction

Approximately 50 million people globally live with dementia. This number is predicted to increase by about 10 million every year (WHO, 2018). Globalization leads to an increase in Europe of the number of older people born in other countries. The increasing number of older immigrants means that dementia-related illnesses within this population have become increasingly common, and the demand for adaptable dementia evaluation, treatment, care and support is expected to increase significantly (Nielsen, 2012; Nielsen et al., 2015). In Sweden, immigrants constitute about 20% of the population (SCB, 2019). These include people originating from around 200 different countries with heterogeneous educational, linguistic and cultural backgrounds. Previous research has shown the range of challenges in offering dementia care to these people (Daker-White et al., 2002; Dilworth-Anderson & Gibson, 2002; Mukadam et al., 2011; Nielsen et al., 2015; Nielsen, Vogel, Phung, et al., 2011a; Plejert et al., 2015; Segers et al., 2013; Wändell et al., 2019).

In current practice, dementia is diagnosed clinically through cognitive testing, physical and psychological examinations, blood and spinal fluid testing, neuroimaging, mapping of the patient’s practical abilities, activity assessment, medical history, interviews with next of kin and questions concerning their perceived quality of life (Winblad et al., 2016). Dementia assessment is a social situation that requires well-functioning communication and interaction between those involved (Ardila, 2005). Accurate evaluation and mapping of patients’ cognitive abilities and limitations is of great importance with respect to the diagnosis. It is also important and crucial for planning person-centered care and caregiving and the support that can be offered to the person with dementia and their relatives (Dubois et al., 2016; Nielsen, Vogel, Phung, et al., 2011a; Winblad et al., 2016).

In many conversations conducted daily within the hospital and healthcare sector, there is a requirement for the participation of an interpreter to allow accurate communication between the patient and the healthcare professionals (Gustafsson et al., 2013; Plejert et al., 2015; Fioretos et al., 2020; Haralambous et al., 2018). When barriers in communication occur between patients and healthcare professionals, the risk of misdiagnosis increases (Hadziabdic & Hjelm, 2014). Linguistic difficulties and cultural differences between the healthcare professionals and the patient may affect the reliability and quality of the results of dementia assessment and diagnosis (Ardila, 2005; Daker-White et al., 2002; Haralambous et al., 2018; Majlesi & Plejert, 2018; Naqvi et al., 2015; Nielsen, Andersen, Kastrup, et al., 2011c; Nielsen, Vogel, Phung, et al., 2011a; Nielsen & Waldemar, 2016; Plejert et al., 2015). When the assessment is conducted using an interpreter, it is important that the interpreter has proficient linguistic skills in both languages being spoken (Eklöf et al., 2014; Fioretos et al., 2020; Gustafsson et al., 2013). It is as well important that the interpreter has formal education. Interpreters who lack interpreter education may negatively affect the interaction between the patient, healthcare professional and interpreter (Fatahi et al., 2010; Plejert et al., 2014).

### The Role of the Interpreter

The use of interpreters in Sweden is regulated by laws such as the Administrative procedure act, *Förvaltningslagen*, 13§ (2017:900), which reinforce that when an authority is in contact with someone who does not speak Swedish, they have an obligation to use an interpreter and to translate documents if needed. Since 1980, the document *Good Interpreting Practice* (*God tolksed)* has defined the occupational ethical guidelines and practical role of interpreters. *Good Interpreting Practice*, produced by the Legal, Financial and Administrative Services Agency (Kammarkollegiet) provides general guidelines that are applicable to all interpreters regardless of their qualifications (Kammarkollegiet.se). In *Good Interpreting Practice* it is specified that the interpreter should interpret everything that is said, interpret in the first person, be neutral and impartial, conform to the laws of confidentiality and maintain professional secrecy. *Good Interpreting Practice* formulates how a proficient interpreter should act and how an interpreter-mediated meeting should be conducted. In Sweden the Legal, Financial and Administrative Services Agency, is responsible for the authorization of interpreters. To become an authorized interpreter, the applicant must undergo the Legal, Financial and Administrative Services Agency’s qualifying examination. After the interpreter is authorized, the interpreter may progress further and become an authorized healthcare interpreter (ibid.). However, in Sweden there is currently a lack of authorized and qualified interpreters, and of interpreters with basic education (Fioretos et al., 2020). This may lead to serious consequences for patient safety, patient integrity, and safe and effective care.

### Dementia Assessment of Non-native Swedish Patients

Cognitive screening instruments used during the dementia assessment can also affect the communication and interaction between the patient and healthcare professionals. Previous research showed that linguistic, cultural and educational background can affect the results of a dementia assessment. The commonly used screening instrument is not always adjusted for use when assessing patients born in other countries (Ardila, 2005; Daker-White et al., 2002; Naqvi et al., 2015; Nielsen et al., 2015; Nielsen, Vogel, Phung, et al., 2011a; Nielsen & Waldemar, 2016; Plejert et al., 2015; Sagbakken et al., 2018). RUDAS, The Rowland Universal Dementia Assessment Scale, is a cognitive screening instrument that is less affected by the patient’s linguistic, cultural and educational background (Nielsen & Jorgensen, 2020; Nielsen et al. 2017). This test is currently used in several countries including Sweden.

Studies conducted in Denmark showed a higher percentage of misdiagnosis during assessment of patients born in other countries because of difficulties during the dementia evaluation process (Nielsen et al., 2018; Nielsen, Vogel, Riepe, et al., 2011b, Nielsen, Andersen, Kastrup, et al., 2011c). Communication is a basic requirement for well-functioning health and medical care (Hadziabdic et al., 2010), and it is necessary for achieving person-centered care (McCormack & McCance, 2006; Plejert et al., 2014). Previous studies describe the difficulty of identifying symptoms of cognitive decline when there are linguistic barriers between the patient and healthcare professionals. This complicates the establishment of a diagnosis (Daker-White et al., 2002; Haralambous et al., 2018; Plejert et al., 2015). There is a need to identify the underlying causes of inadequate dementia assessment in patients born in other countries and the challenges and difficulties that may arise when performing an interpreter-mediated dementia assessment. There is also a requirement to develop strategies and guidelines to improve dementia assessment and diagnosis in patients born in other countries (Nielsen, Andersen, Kastrup, et al., 2011c; Plejert et al., 2015; Stevnsborg et al., 2016). The purpose of this study was to clarify the interaction between patient, healthcare professionals and interpreter during dementia assessment, focusing on how the interpreter may influence the outcome of the evaluation.

## Materials and Methods

This was a qualitative study with an ethnographic design comprising observation through audio and video recordings of 19 interpreter-mediated dementia assessments. This method allows enhanced insight and understanding of how people interact with each other in different contexts (Lambert et al., 2011; Polit & Beck, 2013; Savage, 2006). By performing observations in a natural environment, the method provides insight into how the individuals relate to each other, what they say, and those challenges, obstacles or possibilities that emerge that affect their interaction in the current context (Atkinson & Pugsley, 2005; Hammersley & Atkinson, 2007; Lambert et al., 2011; Polit & Beck, 2013). In addition to the recordings, interpreters and healthcare professionals (doctors and nurses) were interviewed by authors 1 and 2 using semi-structured interview questions about how the interviewee experienced the interaction during the meeting. The study was carried out at a memory clinic in southern Sweden. The dementia assessment was split into two parts and began with a visit to a nurse followed by a visit to a doctor. The study required contacts and coordination with and between the interpreter agency and the corresponding clinic. The study was conducted with the aim that it should have minimal effect on the clinical process (e.g., Hammersley & Atkinson, 2007). So as not to infringe upon the duration of the patient’s visit, the interpreter was asked to arrive fifteen minutes before the patient. The interpreters received the written study information at the same time as the order for an interpreter was made through the interpreter service. The interpreter was briefed about the study and signed a note of consent before the arrival of the patient. In total, 19 assessments were documented using audio and video recording.

### Data Collection

All patients arriving at the memory clinic for dementia assessment from 2015 to 2017 who needed an interpreter were asked to participate in the study. A translation of the study information in the relevant language, made by a translation agency, was presented to the patient after it was conveyed orally by the interpreter. The study information indicated that participation was voluntary and that participants could withdraw at any time, and it described the confidentiality requirements for presenting the results of the study (Polit & Beck, 2013). The audio and video recordings were made using a tape recorder placed at the table and two cameras placed in different locations in the room. The length of each recording was between 40 and 90 min. The data collection also included interviews with interpreters and healthcare professionals, which were conducted in association with every observation. To conduct the study, another person with linguistic mastery of the other language and Swedish was given access to the audio recordings. The persons with linguistic mastery were professional linguists with positions at different Universities in Sweden and one was working as an interpreter in another part of Sweden. These persons transcribed and translated what was being said verbatim into Swedish. The person with linguistic mastery had no access to the video recordings.

The healthcare professionals (doctors and nurses) participating in the study had from one to over 30 years of experience working with dementia assessment, and they had varied experiences of using an interpreter. Seven of ten participating healthcare professionals were women. All healthcare professionals had Swedish as their mother tongue except one, and this person spoke fluent Swedish. The languages spoken by the patients screened were Arabic, Bosnian, Finnish, Greek, Macedonian, Persian, Spanish, Somali and Hungarian. Authorized healthcare interpreters were requested for all sessions. Despite this, only two of the interpreters participating in the study were authorized interpreters or authorized healthcare interpreters. Four of the interpreters had completed the basic interpreter education given in Sweden and the remaining interpreters had no formal interpreter education. Four of the interpreters had no previous experience of interpreting at dementia assessments. Among the interpreters who participated in the study, nine were woman and six were men. The patients participating in the study had varying educational backgrounds. Four of them had less than four years of schooling, eight had 5–12 years of schooling and two had more than 12 years of schooling. The patients who participated in the study included eight women and six men.

### Data Analysis

During ethnographic studies, data collection and analysis occur concurrently (Carlson et al., 2011; Hammersley & Atkinson, 2007). The data were analyzed during the verbatim transcription of recorded data while repeatedly listening to the audio recordings and viewing the video recordings (Hammersley & Atkinson, 2007; Polit & Beck, 2013). The material was transcribed by author 1 and 2, who made reflective notes as they were viewing and analyzing the recorded material and during the transcription of the audio recordings (Carlson et al., 2011; Hammersley & Atkinson, 2007). Viewing and listening to the recordings repeatedly allowed the identification of several details that were important to allow a nuanced understanding of the interaction of the participants (Carlson et al., 2011; Hammersley & Atkinson, 2007). Because the participants spoke languages other than Swedish, the analysis process was based on the video recordings and the translated transcriptions of the audio recordings.

The analysis method was inductive and adhered to the principles of the constant comparative method in conformance with Hammersley and Atkinson (2007) and Glaser and Strauss (1967). The analysis method was considered adequate for an unconditional analysis of data to identify and define what was important for the context that was observed, and for the persons that observed (Polit & Beck, 2013).

The analysis began with a meticulous review of all the gathered material. Patterns in the material that related to parts of the dialogues between the participants that were deemed relevant to the study’s purpose were marked. These patterns formed the basis for the analysis (Hammersley & Atkinson, 2007). The next step was to condense the patterns to shorten the text, making it more easily manageable (ibid), then the condensed text was abstracted through encoding. The encoded texts were compared with each other and encoded texts that included similar concepts were classified. Many encoded texts with similar content constituted a category that comprised several subcategories. The content of the categories and subcategories were compared with each other to establish the compliance of all encoded texts. The intention was for the content of all categories and subcategories to be clearly defined, and that encoded texts, subcategories and categories be distinct from each other (Glaser & Strauss, 1967; Hammersley & Atkinson, 2007). It was important that the researchers were aware of their own impact during both the observations and the analysis. Reflections about the influence the researchers might have during the analysis process were important in order to maintain the trustworthiness of the results (Davies, 2008; Hammersley & Atkinson, 2007). It was also important that the authors 1 and 2 analyzed the material independently. Authors 1 and 2 participated in the analysis and the establishment of categories and subcategories.

### Methodological Discussion

The ethnographic method of observations through video and audio recordings presented an opportunity to study and receive a deeper insight into what happened during the interactions between the patient, healthcare professional and interpreter during a dementia assessment (Polit & Beck, 2013). The video observations were similar to personal observations, but without the observer affecting the participants by their presence (Eidevald, 2015; Rindstedt, 2013). It is possible that the video recording affected how the participants acted and did not capture how the participants usually act, because they were aware that what they said and did was being recorded and analyzed (Eidevald, 2015). On the other hand, this awareness may have made them perform at their best during the meeting and show the quality they strived for (Eidevald, 2015). Interviews conducted after every observation complemented the picture of the interaction that was observed.

The first author is experienced in conducting dementia assessment using an interpreter. Continuous reflections and discussions with the coauthors, who are of different professions, was of importance during the analysis process (Carlson et al., 2011; Davies, 2008 ; Hammersley & Atkinson, 2007).

The person with linguistic mastery who transcribed and translated what was said in the other languages had some comments about the cultural aspects affecting the communication that were important for the analysis of the material. Aspects of culture and gender, which could affect the interaction between the participants, have not been discussed at length in this article. By describing the context, data collection and analysis process, and by comprehensively presenting the results using quotations from the observations, we aimed to increase the ability of the reader to familiarize themselves with what was presented and be able to make their own interpretations to judge the credibility, reliability and generalizability of the findings (Eidevald, 2015; Hammersley & Atkinson, 2007).

### Ethical Considerations

Approval by the Swedish ethical review authority, Etikprövningsmyndigheten, was obtained for this study according to the Swedish law concerning evaluation of the ethics of research pertaining to human beings (2003:406), with approval number 2014/492. It was important that researchers ensured that the patients understood the information presented about the study. If the patient or healthcare professional in charge of the meeting had any hesitation, the video and audio recording was discontinued. When quotations was presented, a code was used as a reference.

## Results

The study showed that there were alterations in what was communicated when using an interpreter. Several difficulties in communication occurred between patient, healthcare professional and interpreter. All participants affected the communication and the interaction in different ways. In this study we are focusing on the interpreter and the influence the interpreters might have on the outcome of this interaction. These difficulties were often due to the interpreter’s language skills in Swedish and the other language, and because the interpreter did not adhere to the guidelines of *Good Interpreting Practice*. Information, instruction, questions and answers could be changed during the interpretation. Sometimes the interpreter did not interpret everything being spoken and made corrections of their own in what was spoken and communicated. The results of the study were presented from the category of *Meaning and content of what was being said was altered during the interpretation,* and from the five subcategories, *Content being added and removed during interpretation, Linguistic difficulties in Swedish, Difficulty in the other language, The interpreter did not adhere to ethical guidelines, The interpreter’s lack of proficiency affected the patient*. In the examples presented from the observations, healthcare professional is abbreviated as HP, patient as P and interpreter as I.

### Content Being Added and Removed during Interpretation

Interpreters added and/or removed words and sentences, and they made alterations in what the healthcare professionals or patients said. The alterations made by interpreters could change the content of what was being said. These alterations might sometimes improve the communication but often had an effect on the assessment of the patient’s cognitive ability and could have a negative effect on the result of the investigation. One example of this is when the intention of the open question posed by the healthcare professional is altered when interpreted. The question in the example comes from the self-evaluation questionnaire, the Hamilton Anxiety and Depression questionnaire, that was used during the assessment to gauge the existence of depression or anxiety in the patient.

HP: Har du tappat intresset för hur du ser ut? Have you lost interest in your looks?

I:بطلت تهتم كيف شكلك الخارجي You no longer care about your looks.

P:لأ No.

I: Nej. No.

HP: Nej. No. (Example1, observation 9).

Example 1 shows how the quality of the interpretation presented a distorted image of the patient’s perception of their health. During the interpretation, the question was altered to a statement, which lead to a change of the intention and the content of the question. The patient’s response was not to the question being asked, but rather to the statement presented by the interpreter. The result was that the healthcare professional could acquire an incorrect perception of the patient’s feelings.

### Linguistic Difficulties in Swedish

Several of the recorded interpreters had difficulty with the Swedish language, both in understanding Swedish and expressing themselves in Swedish. Some of the interpreters participating in the study, and who were interviewed after the observation sessions, spoke of these difficulties as a problem among other interpreters. The interpreters were of the opinion that it was the responsibility of the interpreter agency to review language skills when hiring interpreters. The interpreters’ difficulties in the Swedish language could cause the healthcare professional to receive an incorrect perception of the patients’ actual cognitive ability.

Medical terms as well as complex and less common words and expressions made the interpretation more difficult for the interpreters. This led to lengthy dialogues between interpreter and patient, misunderstandings, and impaired understanding between the healthcare professional and the patient. This is described in example 2 where the interpreter had difficulty interpreting the words *visual hallucination*.

HP: Har du någonting som du… eh… ser? Har du någon synhallucinationer?

Do you have anything that you…eh…see? Do you have any visual hallucinations?

I: Дали понекогаш гледаш некои слики, нешто да ти се преметнува?

Do you see any pictures/paintings sometimes, does anything show up for you?

P: Слики..., везано, јас имам доста везано.

Pictures/paintings…, woven, I have plenty woven.

I: Hon förstår inte vad, vad du menar. She doesn’t understand what, what you mean.

P: Доста везано имам. I have plenty woven.

HP: Kan du… eh… kan det vara att du ser saker som inte finns där?

Can you…eh...might it be that you see things that are not there?

I: Дали гледаш некои ствари кои не се тука? Дали то ти се јавува некогаш?

Do you sometimes see things which are not there? Does that show up for you sometimes?

P: Не. No.

I: Nej. No.

HP: Nej. Kan du se konstiga saker? No. Can you see strange things?

I: Дали гледаш некои необични ствари? Do you see any strange things?

P: Не. No.

I: Nej. No. (Example 2, observation 7).

In example 2, the interpreter could have explained to the healthcare professional his difficulty in interpreting the words *visual hallucination* and asked the healthcare professional to rephrase the question, but instead the interpreter tried to explain it to the patient. The interpreter also did not interpret what the patient said. There was a long dialogue before the patient understood the question and answered.

In example 3, the healthcare professional asked the patient to name as many different animals as possible in a minute. This posed no difficulty for the patient, but the interpreter had difficulty interpreting several of the animals mentioned by the patient. Instead of explaining this to the healthcare professional, the interpreter tried to make up words, which gave a completely distorted image of the patient’s linguistic ability and memory capacity. The interpreter was told to write the named animals down in order to not disturb the patient.

HP: Okej, du har en minut på dig, säg så många djur du kan. Okay, you have one minute, name as many animals as you can.

I:انت قل اكثركل ما تعرف كل الحيوانات اللي خلال دقيقة تعطيني حيوانات اكثر.

You are to say all animals you can within one minute. Give me more animals.

P:ابقار واغنام او Cows and sheep and… او بعير وجاموس او غزلان وه .. سلاحف شونو هي هاي اكتفي بهادي and camel and buffalo and gazelle and ... turtles – and what – that that’s enough with these.

I: Eh… det räcker med dom här… Han säger det räcker med dom.

Eh… that’s enough with those … He’s saying it’s enough with those.

HP: Du kan fortsätta. You may continue.

I:واصل واصل عندك دقيقة Continue continue you have one minute.

P:ايه اسد نمر ابن اوى .. هادا كثير eh lion tiger jackal... isn’t that a lot.

I: Det räcker. That’s enough.

HP: Ja. Okej. Yes. Okay.

I: (Counting the animals he wrote down). Nio. Nine.

HP: Hm… många djur. Jag ber tolken översätta orden. Hm… many animals. I’ll ask the interpreter to translate the words.

I:انا نتظر المترجم يترجملك اياها انا بترجمهم بالسويدي We’re waiting for the interpreter to translate them. I’m translating them to Swedish.

P:ايه Eh…

I: Eh… ja. Det är lamm… ko… eh… det måste… den stora… ögon… ögonhörning. Det här… liknar…

Eh…yes. It’s lamb … cow…eh…it must…the big…eyes…eyenoceros. This one…looks like…

HP: Noshörning. Rhinoceros.

I: Eh… flodhäst, flodhäst… eh… kamel… gasell… eh… djur utan ben vad heter dom… kryp, krypdjur.

Eh… hippopotamus, hippopotamus…eh…camel… gazelle…eh… animals without legs what’s their name…crawl, crawlanimal.

HP: Orm. Snake.

I: Krypdjur. Krypdjur… det… är andra sortering alltså och lejon… tiger… och en… varg.

Crawlanimal. Crawlanimal…it… is other sorting so and lion…tiger…and a…wolf.

HP: Hm… Bra. Hm… Good. (Example 3, observation 11).

Example 3 shows that the interpreter had difficulties to interpret the animals mentioned by the patient. Thus, the patient’s answer became unclear, awkward, and weird via the interpretation process. This could present to the healthcare professional an incorrect image of the patient’s cognitive ability. Similar problems occurred in several of the recorded assessments, where the healthcare professional’s evaluation of the patient’s cognitive abilities could be affected by the interpreter’s linguistic knowledge. It was not possible for the healthcare professional to know when the interpreter was having difficulty interpreting, which could be mistaken for cognitive difficulty in the patient. The interpreter participating in the observation quoted above, in example 3, mentioned in the subsequent interview that problems had occurred during interpreting but that these were eventually resolved when he finally remembered a word he had forgotten. However, the interpreter did not mention any of this to the healthcare professional. The interpreter also did not understand how this, or similar situations could affect the healthcare professional’s image of the patient’s cognitive ability.

### Difficulty in the Other Language

Several of the interpreters recorded had difficulty in the other language. This could be because they did not have a satisfactory mastery of the other language or because the interpreter and patient were speaking in different dialects. These difficulties resulted in the interpreter being unable to assist the patient and healthcare professional in understanding each other, which in turn could lead to erroneous evaluation of the patient during the dementia assessment. The healthcare professional could mistakenly perceive that the patient was having difficulty understanding questions or instructions when, in reality, it was the interpreter having difficulty understanding and interpreting in a satisfactory manner. In example 4, the healthcare professional asked a question from the cognitive screening instrument the Mini-Mental State Examination, which was intended to evaluate orientation in time (Folstein et al., 1975). The interpreter had difficulty interpreting the question, which resulted in the patient having difficulty answering. The question became difficult and complicated for the patient to understand because of the lack of competence of the interpreter and the poor quality of the translation. Example 4:

HP: Vilken veckodag är det? Which weekday is it?

I: Wiiggu waa wiiggee? Wiiga aan ku jirno? Which week is this week? Current week?

P: Maxay tahay wiigaga? Maxay kala yihiin? Wiiggagu maxay yihiin? Wiigagu maicnaheedu waa maxay? Wiigagga waa maxaay? What is weeks? What’s meant by weeks? What mean by weeks? What means by that?

I: Eh, vad menar du när du säger… Eh, what do you mean when you say…

HP: Är det måndag, tisdag, onsdag, torsdag eller fredag? Is it Monday, Tuesday, Wednesday, Thursday or Friday?

P: Maalmaha. Days?

I: Een, maalmaha. Dagar. Days. (Example 4, observation 2).

In the task above, example 4, it became difficult for the patient to answer because of the interpreter’s lack of proficiency of the other language. However, when the patient realized what the interpreter meant, the patient corrected the interpreter. The healthcare professional who thought that the patient did not understand the question attempted to rephrase the question, which could reduce the patient’s score in the examination, because presenting clues is not allowed by the instructions for the examination. Therefore, this will affect the healthcare professional’s assessment of the patient’s orientation in time. In the subsequent interview, the healthcare professional in example 4, said that it felt good with the interpretation because the interpreter spoke fluent Swedish, but did not understand that the interpreter had difficulties in the other language.

Example 5 shows that language barriers arose when the interpreter and patient did not speak the same dialect. This caused misunderstandings and errors in the interpretation. As shown in example 5, it became difficult for the patient and interpreter to understand each other.

P:وعندي سوفان بالرقبة وبالكتف انا ما And I have wear and tear in my neck and shoulder. I don’t have…

I: Hm. Alltså det är därför jag har problem med… eh… knä och axel. Hm. So that is why I have problem with…eh…knee and shoulder.

P:وعندي سوفان And I have tear in my joint (soufan).

I:شو يعني سوفان What is soufan?

P:يعني يطلع صوت Ya’ni, you hear a sound.

I: Hm, hm.

P:هيك يعني يصير الوجع This is how the pain becomes (patient points to neck and shoulders).

I: Jag får… framknä, jag får en ljud. I get… forwardknee, I get a sound.

HP: Förlåt? I’m sorry?

I: Det knä, det knakar. That knee, it creaks.

HP: Hm. Hm.

I: I knäet. In the knee.

HP: Ja. Yes. (Example 5, observation 14).

In example 5, the patient described her pain and the cause of that pain, which was wear and tear in her neck and shoulder. The interpreter, who spoke a different dialect to the patient, did not understand the meaning of the word “Soufan” which is an Arabic phrase pertaining to wear and tear of the joints. The interpreter also translated the words “Belroghbeh and Belketf”, which mean “neck and shoulder”, incorrectly as “knee and shoulder”. This error was never corrected during the conversation, and the information received by the healthcare professional was a deficient and incorrect translation of what the patient said and perceived pertaining to pain in the body. The interpreter in example 5 mentioned in the subsequent interview that he felt uncertain of his interpretation. He and the patient did not speak the same dialect and he was not sure that the patient understood him. “I don’t know if the communication was as it should have been. We did not have the same dialect and I wasn’t allowed to repeat myself. I don’t know if the patient understood me.” (Interview with interpreter from observation 14). This was not brought to the healthcare professional’s attention during the visit.

Several of the interpreters mentioned in their subsequent interview that dialectal differences could occur between them and the patient. Some of the interpreters described dialectal differences as a problem for optimal interpreting, whilst others did not think it had any great effect on the quality of the interpreting. One of the interpreters, who was an authorized interpreter, spoke of the importance of getting information before interpreting about both the context in which the interpretation was to occur, and the dialect spoken by the patient. Another interpreter considered that dialectal differences that often occurred could lead to problems during interpreting but did not feel a responsibility to report this difficulty to the healthcare professional.

I cannot interpret for all dialects or validate that I can interpret everything. Today I was wondering if the patient understood. The patient answered something which didn’t relate to the question but the clinician didn’t say anything and didn’t react (interview with interpreter from observation 9).

The quality of communication between patient and healthcare professional fluctuated throughout the interpreting. At a dementia assessment, the healthcare professional is observing the patient’s linguistic ability via different tests, in addition to conversing with the patient. The results of the study showed that the patient’s actual linguistic skill was not always clear during conversation with the healthcare professional, because it could be affected by the interpreter’s skill and language proficiency.

## The Interpreter Did Not Adhere to Ethical Guidelines

The results of the study showed that the interpreters did not always follow the guidelines described in *Good Interpreting Practice* and this affected the interactions between the patient and healthcare professional during the assessment. The majority of the non-authorized interpreters did not interpret everything being said, they made adjustments and abridgments, and summarized what the healthcare professional or patient said. One of the interviewed interpreters commented as follows:

Sometimes the patient or doctor speak excessively and why should I interpret this? The patient is at the hospital for example because of pain in the stomach, and then everything else, yes, why should I interpret that? (Interview with interpreter from observation 3).

The results of the study showed that the interpreters consciously did not interpret everything being said. Some of the interpreters who participated in the study decided whether what was being said was relevant or not in the context and, thus, at times refrained from interpreting. This decision could affect the interaction, because what was not being interpreted could be an important piece of information for the healthcare professional. The interpreters would also, for example, adjust the language when the patient could not find the correct word or name when they were describing something. The interpreters could also avoid interpreting when the patient repeated a word or a sentence. Some of the interpreters recorded could correct the patients in different situations, for example, when they thought the patient said something unsuitable for the context. This occurred in particular when the interpreter and patient were previously acquainted. According to *Good Interpreting Practice*, the interpreter should always state whether there exists a bias, in which situation they should cancel the interpreting assignment. In the cases where bias occurred, the interaction and assessment could be affected, because the interpreters would interfere in the matter at hand. They could not be neutral and impartial, according to *Good Interpreting Practice*, and would act more or less as the patient’s lawyer or negotiator rather than as an interpreter. They would also question what the patient or healthcare professional said, or fill in when the patient was having difficulty on their own. These interpreters would also describe the patient’s perceptions, problems and difficulties in their own way. Example 6 is part of a conversation between the interpreter and the patient when the healthcare professional had left the room for a few minutes to pick up something she had forgotten. The interpreter, who knew the patient privately, thought he should not complain over his situation.

P: […] што да правиме бре, X. Толку знаеме.

What are we to do now. We can so much.

I: Шеј, остарефвме, болести си идат сега, не треба да се жалиме, добри сме...

Listen, we’ve become old, now illnesses come, we shouldn’t complain, we’re well, it’s good… (Example 6, observation 7).

After this conversation, between him and the interpreter, the patient was silenced and did not tell much more about himself.

According to *Good Interpreting Practice*, the interpreters should interpret in the first person; however, the interpreters did not always adhere to this regulation, but rather switched between first person and speaking of the patient or healthcare professionals in the third person. It was more common to depart from the first person when the interpreters did not quite understand what they were to interpret, or when the interpreters became uncomfortable in an interpretation situation. Not interpreting in the first person also occurred at times when the healthcare professionals were not sufficiently clear in what they said, and the interpreter attempted to fill in and make what the healthcare professionals said more comprehensible for the patient. The interpreter not interpreting in first person would lead to the patient beginning to talk about the healthcare professional in the third person.

HP: Då ska du få rita igen fast att du inte tycker om det…så ska du rita en klocka. (Litet skratt). Now you are to draw again even though you don’t like it…so you are to draw a clock. (Minor laugh).

I: Haa. Adigoon aan ka helin oon ogsoonahay bay ku tiri bal. midi ugu danbeysay aan sawirno. Yes Even if you don’t as I know like it, she told you to draw for the last time.

P: Oo maxay ah? And what is it?

I: Iyadaa soo bixineysa. She’s about to bring it up. Och vad det är för, vad ska han rita? And what is it, what’s he to draw?

HP: Rita en klocka och sätta ut siffrorna. Draw a clock and label it with numbers.

I: Waxaad sawirtaa saacad waxaadna ku dhex qortaa een, xisaabti ku dhex qor saacadaha. You are to draw a clock and you put numbers on the clock.

P: Waa adagtahay dheh. Tell her it’s difficult.

I: Det är ju svårt. It’s difficult.

P: Waa adagtahay. It’s difficult.

HP: Hm. Hm. (Example 7, observation 2).

In example 7, it seemed as the interpreter became uncomfortable with the situation when the healthcare professional wanted the patient to do something that the patient was not interested in. The patient had several times previously told the staff that he was not good at drawing. The interpreter told the patient to follow what the healthcare professional said or did as opposed to interpreting what the healthcare professional said in the first person. The quotation shows how the patient started to speak about the healthcare professional as opposed to with the healthcare professional.

### The Interpreter’s Lack of Proficiency Affected the Patient

Some of the patients recorded would become more uncertain when they did not understand the interpreters. Questions asked to the patient would become more complicated and stressful when the patient did not understand or could not make themselves understood. The interpreter’s interpretation and choice of words could lead to patient misunderstanding. In example 8, the healthcare professional explained to the patient that the tests performed during the meeting were part of the dementia assessment.

HP: Så det är en del i utredningen. So it’s a part of the assessment.

I:هذا جزء من التحقيق This is a part of the interrogation.

P: تحقيق شو عندنا مركز شرطة ها ها What interrogation. Is there a police station here ha ha ha.

I: Utredning hos polisen eller? Assessment with the police or?

P:ها مركز شرطة Ha police station.

I: Han säger förlåt mig jag bara tolkar den utredningen…

He’s saying forgive me I’m just interpreting the assessment.

HP: Ja. Yes.

I: …och det användes på fel sätt. …And it was used incorrectly.

HP: Ja, minnesutredningar. Yes, memory assessment.

I:دراسة الذاكرة .. حتى يعمل جزء من دراسة للذاكرة Study of the memory to make a part of the study of the memory (Example 8, observation 11).

In the situation in example 8, the word “assessment” was interpreted as “interrogation”. The patient was confused and questioned what this meant. Sometimes the patient’s irritation would affect their willingness and motivation to continue being a part of the assessment. Some of the patients were skeptical of the interpreter and wondered whether the interpreter was really able to perform her/his job, whether he/she was interpreting everything, whether he/she was interpreting correctly, or whether the interpreter really understood the healthcare professional. During the subsequent interview, the interpreter participating in the observation above was pleased with his work as an interpreter and thought the interpreting had gone well. The interpreter never mentioned the patient’s irritation, which appeared several times during the course of the evaluation.

The example below is from one of the observations where the interpreter did not have much experience in interpretation and was not fluent in the other language. The patient showed his dissatisfaction both to the interpreter and to the healthcare professionals several times during the evaluation. Finally, the healthcare professional requested the patient to name as many different animals as possible in one minute. The patient stated several animals but became uncertain of the interpreter’s ability to interpret. The patient then asked the interpreter: *P: Dhurwaay aa la garan. Dhurwaa ma taqaan? I know hyena. Do you know hyena? (Exampel 9, observation 2).*

This patient expressed his dissatisfaction several times during the cognitive assessment. He corrected the interpreter when the interpreter was unsure or had difficulty in the other language. He questioned the healthcare professional when communication was unclear. The interpreter did not interpret when the patient said something about his dissatisfaction, and this was not noticed by the healthcare professional either. This patient did not return for further investigation.

## Discussion

In summary, the study showed that the interpreter’s knowledge of Swedish and the other language, as well as their professionalism and approach to the guidelines governing their work, affected the interaction and communication during dementia assessments. This affected and, thus, could impair the quality and reliability of the assessment. This could mean an increased risk that the healthcare professional misjudge the patient’s cognitive abilities, as well as their physical and mental state of being.

Previous studies suggested that the healthcare professionals perceive that they are providing better quality of care when they use a professional interpreter (Bauer & Alegria, 2010; Eklöf et al., 2014; Granhagen Jungner et al., 2019; Haralambous et al., 2018; Karliner et al., 2007; Krupić et al., 2019; Silva et al., 2016). The results of the present study revealed several challenges and problems that could arise despite the use of professional interpreters hired through an interpreter agency. There is a risk that the healthcare professional experienced a sense of security that was not well-founded when using interpreters hired from interpreter agencies. The language knowledge and educational background of the interpreters participating in the study varied, and the majority of the interpreters were not authorized and had no formal interpreter education. The healthcare system should be more attentive to this and enforce on interpreter agencies their requirements for language knowledge and interpreting training of the interpreters being used.

Dementia assessment is a special context in which interpreting requires more concentration and awareness by the interpreter than many other types of interpreting situations within healthcare (Haralambous et al., 2018). One of the most common symptoms of dementia is difficulty in speaking and finding the correct word (APA, 2014). During the evaluation and examination, the language difficulties of the interpreter could have presented a distorted perception of the patient’s physical and psychological health in addition to their linguistic ability. Accurate interpretation and ensuring that everything being said is interpreted is crucial to allow the healthcare professional to be able to observe and judge any difficulties for the patient. The results of this study showed that this often did not operate as it should. The results of this study confirm Wadensjö (2018) who described that the interpreters could add or expand information to the original utterance. The interpreters could reduce or not interpret everything being said. Also, they did not always interpret the original utterance and could say something which was not part of the interpretation (Wadensjö, 2018).

When the interpreters did not interpret accurately everything being said, altered what was being said, and/or had difficulties in Swedish and/or the other language, the healthcare professional’s image of the patient’s condition could be affected. It became difficult and often impossible to conduct a fair evaluation and assessment of the patient’s language and other cognitive abilities. Because these assessments are used as the basis for formulating a diagnosis, this may lead to misjudgments. The results of our study confirm the findings of Majlesi and Plejert (2018) and Plejert et al. (2015), who reported that communication difficulties during dementia assessment conducted via an interpreter may lead to misjudgments and misdiagnoses.

There were also several occasions where the patient and interpreter spoke the same language but with different dialects. This led to difficulties in the ability of the patient and interpreter to understand each other and, therefore, could affect the assessment. This information was not brought to the attention of the healthcare professional during the meeting but was rather mentioned by the interpreters during their interview after the meeting. This is a clear breach in Good Interpreting Practice which says that the interpreter must resign from the assignment if he or she is unable to perform it satisfactorily.

During the post-meeting interview with the interpreters, some of them voiced concern over these differences in dialect. But there were also those of the non-authorized interpreter who did not think it had affected the communication with the patient; this differs from the conclusions drawn from the analysis of the material. Some of the interpreters thought this was the responsibility of the healthcare professionals, and that it was something that healthcare professionals should consider when booking an interpreter. The results of this study present important findings about using an interpreter and suggest that healthcare facilities should establish guidelines for using and booking interpreters. It is also important to have routines for establishing that the interpreter in the meeting has relevant qualifications.

It is crucial for optimizing communication between the patient, interpreter and healthcare professional that the healthcare professional should have full information about the patient’s native language and dialect before booking interpreters. Information about language may be lost in the booking procedure so it is crucial for the healthcare professional to double check that it is correct with the patient and the interpreter at the meeting.

Alterations in content and meaning made by the interpreters could also be dependent on their approach. Because several of the interpreters, with no formal education or with basic interpreter education, could ignore their role and the power they might have, and they did not adhere to the guidelines governing their profession. Some of the interpreters observed actively chose not to interpret everything. This choice has been confirmed by other research that described the issue from the healthcare professional’s perspective and that claimed that the healthcare professional sometimes perceived that everything said was not being interpreted, or not in the way it was stated (Haralambous et al., 2018; Sagbakken et al., 2018). Lundin et al. (2018) also emphasized that it was important for improved quality of care that interpreters respect the ethical guidelines governing their profession.

The interpreters observed in this study would, instead of interpreting everything being said, answer the patient directly. This created a dialogue between the interpreter and patient without the participation of the healthcare professional (Wadensjö, 2018). These dialogues could sometimes improve the communication or be the interpreter’s attempt to help the patient understand, which Plejert et al. (2015) describe as repair sequences. But yet again, important information for the healthcare professionals or patients could be lost or altered when the interpreters explained and communicated with the patients on their own. Information and instructions could be altered and communicated as the interpreter saw fit. This result was consistent with those of other researchers who described interpreters who, in wanting to facilitate communication between patients and the healthcare professional, sometimes made linguistic alterations so that the healthcare professional received a “good” or more suitable answer from the patients. During dementia assessments, these alterations may cause misleading results and invalidate the tests (Majlesi & Plejert, 2018; Nielsen, 2012; Plejert et al., 2015).

The results of the study showed that several of the interpreters being observed did not understand certain parts of the assessment. This was because they had never interpreted in similar contexts and had not received information about the meeting and what was to happen; consequently, their interpreting was affected. Majlesi and Plejert (2018) and Plejert et al. (2015) claimed that interpreting can be affected by the context in which the interpreter is interpreting and by the individuals participating and interacting in the meeting. Previous research and the results of this study suggest that it may be of great importance to present the interpreters with information and knowledge about the assessment and the rules that govern the testing before the evaluation begins. This would optimize the basis for interpreting and enhance the quality and reliability of the dementia assessment. These results are consistent with those of Lundin et al. (2018), who emphasized the importance of interpreters’ awareness and knowledge of the context in which they are interpreting.

The results of the study showed how some of the interpreters affected their relationship with the patient and also the relationship between the patient and healthcare professional because of language difficulties and by not adhering to the guidelines governing their profession as an interpreter. This could have significantly impacted how the patient performed during the meeting and, thus, affected the dementia assessment. The present study showed that when the patient and interpreter had difficulty understanding each other, this affected the patient’s sense of trust and comfort when interacting with the interpreter and healthcare professional. One example of this is when the patient in example 9 questioned the interpreter’s competence and wondered if the interpreter had sufficient capacity to interpret what the patient said. That patient did not return for the next visit to continue the investigation. This might be because the first visit was a bad experience for him where he lost confidence and therefore he did not want to come back.

Some patients who participated in the study were suspicious that the interpreter in their session was not interpreting everything being said correctly. Some of the patients participating in this study, as in example 9 admonished the interpreter or questioned the healthcare professional when the interpreting was not satisfactory. This discontent was later directed towards the healthcare professional and would affect the patient’s willingness to participate during the dementia assessment, as in example 9. This finding is supported by those of Eklöf et al. (2014), who claimed that inexperienced and incompetent interpreters could complicate and aggravate the relationship between patients and healthcare professionals. Fatahi et al. (2010) and Lundin et al. (2018) also emphasize that the competence of the interpreter and the patient’s trust in the interpreter are essential to the quality of the communication. The results of our study are also consistent with those of Silva et al. (2016), who showed that when interpreting and the use of an interpreter did not occur in an adequate and satisfactory manner, consequences such as a lack of patient participation and a lack of understanding of their diagnosis, symptoms and treatment can arise.

Dementia-related illnesses are incurable and existing medical treatments focus on alleviating symptoms. This enhances the importance of a reliable assessment and diagnosis to offer supportive interventions for the patients and their relatives, in cooperation with whom the treatment and care should be planned. This requires a good relationship and cooperation with the patient, and it is possible only when there is trust and confidence between the patient and healthcare professional. This trust is at risk when communication malfunctions during the assessment process and can lead to the patient mistrusting the healthcare professional. Rothlind et al. (2018), who have studied intercultural healthcare meetings, report that when communication malfunctions, it can cause reduced trust from the patient not only towards the people the patient has directly encountered but also towards the entire healthcare system.

The number of people requiring interpreting during dementia assessment will continue to increase in Sweden and in other European countries. However, in Sweden there is a lack of educated professional interpreters and an even greater lack of authorized healthcare interpreters. When communication cannot be ensured, the quality and security of the healthcare provided can be called into question.

The study identified a number of challenges and difficulties that can arise when the dementia assessment is conducted via an interpreter. How the healthcare professional monitors an evaluation conducted using an interpreter is of great importance for the quality and reliability of the assessment, because they are responsible for the meeting. Therefore, it is important in subsequent studies to delve deeper into how healthcare professionals interact with patients via an interpreter, and to determine which factors can affect this interaction and, ultimately, the quality of the assessment.

## Conclusion

The study showed that when the meaning and content of the language, information and instructions are altered by the interpreter, there are risks of misjudgments about the patients’ cognitive ability and the dementia assessment might not be reliable. It is of great significance to the quality of care that the interpreter possesses sufficient skills in both languages and is trained in interpreting. The consequence of language difficulties between patient and interpreter can affect the trust and confidence the patient has in healthcare, which consequently can negatively affect the patient’s cooperation during the assessment. It is also of great importance that the interpreter has a professional approach and adheres to the ethical guidelines governing the interpreters’ profession. Otherwise, the assessment may not be reliable, and the evaluation of the patient’s cognitive ability can be misleading.
